# Medical Society of the County of Erie

**Published:** 1908-06

**Authors:** Franklin C. Gram

**Affiliations:** Secretary


### SOCIETY PROCEEDINGS.

### Medical Society of the County of Erie

Reported by FRANKLIN C. GRAM, M.D., Secretary.

THE regular quarterly meeting of the Medical Society of the County of Erie was called to order at 4 o'clock p. m., Monday, April 20, 1908, in the rooms of the Buffalo Society of Natural Sciences, Buffalo Library Building. In the absence of the president, Dr. Edward Clark, the first vice-president, Dr. Charles A. Wall, took the chair.

The secretary read the minutes of meeting held January 13, 1908, which were approved as read. On motion of Dr. Bennett, the society then went into executive session, and all persons who were not members were requested to withdraw. The secretary then read the minutes of the meeting of the Council held January 23, 1908, those of meeting held April 9, 1908, and the minutes of meeting of the council held April 13, 1908.

On motion of Dr. Grosvenor, the various items in the council proceedings were considered seriatim. Dr. Grosvenor moved that, in place of the council's recommendation regarding the question of salary for the secretary and treasurer, an amount of fifty dollars ($50.00) be appropriated to each for the current year.

Dr. Briggs moved to amend by approving the action of the council appropriating certain compensation to the secretary and treasurer, also striking out the word "annual," and that, in future, the amount of salary be referred to a committee of five (not members of the council) to make its recommendation at the next annual meeting. Both amendments were lost. Dr. Grosvenor then moved to disapprove the action of the council relative to the question of salaries. Carried. Dr. Rochester moved that the council give serious attention to the question of salaries of secretary and treasurer and that their necessary expenses be paid. Carried.

On motion of Dr. Grosvenor, the action of the council relative to sending out all notices with two-cent postage was approved, and it was decided that all future notices be sent in a similar manner. On motion of Dr. Briggs, the appropriation made by the council to the membership committee was approved. The balance of council proceedings were then approved.

A communication was received from Dr. Dewitt G. Wilcox of Buffalo, relative to a proposed visit of Dr. G. W. Arnold of Minneapolis, Minn., who intends coming to Buffalo and several other places on a lecture tour, in which he deals with medical institutes, various advertising specialties such as "lost manhood," and the like. On motion of Dr. McKee, the communication was referred to the Board of Censors.

A report made by Dr. A. L. Benedict, chairman of the special committee, and which was intended for presentation at the previous meeting of the society, was referred to the committee on membership. An invitation was received from the Universal Club to attend a debate for the same evening. On motion, it was received and filed.

The Censors, through Dr. Rochester, reported progress. Dr. E. E. Snow, of Batavia, president of the Eighth District Branch, was introduced to the society and made a few remarks.

Dr. A. T. Lytle, chairman of the auditing committee, reported that his committee had examined the books and accounts of Treasurer Greene for the year 1907, and found them correct. The report was approved.

Dr. McKee, chairman of the committee on membership, presented a report of that committee. Dr. Grosvenor moved that the names of applicants be considered individually. Carried. The following applicants for membership were then considered and each elected separately by ballot.

Charles A. Bentz, 84 Orange St., Buffalo; Herman D. Andrews, 399 Delaware Ave., Buffalo; William A. McFarlane, Springville; John G. W. Knoll, 1067 Ellicott St., Buffalo; Eugene R. Linklater, 1540 Main St., Buffalo; Harry M. Weed, 405

Franklin St., Buffalo; Harley L. Atwood, Collins Center; Clarence C. Duchscherer, 247 Forest Ave., Buffalo; John E. Whitmore, 355 E. Utica St., Buffalo; George A. Sloan, 194 Fourteenth St., Buffalo; Loretta L. Knappenberg, 112 Triangle St., Buffalo; Dewitt G. Wilcox, 597 Elmwood Ave., Buffalo; William H. Billings, 1272 Main St., Buffalo; Cora B. Lattin, 146 Humboldt Parkway, Buffalo; George McK. Hall, 228 E. Eagle St., Buffalo; E. Lowell Marcy, 1335 Jefferson St., Buffalo; Wilford L. Odell, North Collins; Robert C. Mehnert, 1784 Genesee St., Buffalo; Edwin L. Bebee, 437 Franklin St., Buffalo; Thew Wright, 152 Allen St., Buffalo; Francis A. Drake, 409 Connecticut St., Buffalo; Clifford R. Orr, 1093 Ellicott St., Buffalo; Edgar R. McGuire, 470 Franklin St., Buffalo; Thomas J. Walsh, 306 Niagara St., Buffalo; H. W. Lattin, 156 Humboldt Parkway, Buffalo; John Scott McFarland, 1094 Delaware Ave., Buffalo; Jacob W. Bayliss, 592 Spring St., Buffalo; John R. Fairbairn, 155 Allen St., Buffalo; James Hoyt Lewis, 520 Franklin St., Buffalo; James H. Carr, 345 Eagle St., Buffalo; Francis J. Carr, 345 Eagle St., Buffalo; Lynn S. Beals, 119 No. Norwood Ave., Buffalo; Guiseppe Tartaro, 129 Court St., Buffalo; Macy B. Searls, East Aurora; Julius H. Potter, 177 Dearborn St., Buffalo; Fridolin Thoma, 1072 Lovejoy St., Buffalo; Frederick H. Stanbro, Springville; Albert F. Erb, Clarence.

Dr. Lytle moved that orders on the treasurer for three dollars ($3.00) each, which sums shall be deducted from the amount due the State Society as "back assessments," shall be drawn in favor of those who have become or who may become eligible for reinstatement in this society in 1908, and such individuals are hereby declared reinstated in this society; but it is specifically provided that in each such case of reinstatement it must be shown by the books of the treasurer that the individual has paid three dollars ($3.00) into the society as state assessments for 1907, and that in each such case it must be shown by the lists received from the state treasurer that the individual so paying had been dropped from the rolls by the state officers in 1907 for non-payment of dues, and in each such case it must be shown that the individual has become eligible for reinstatement in 1908. Carried

Dr. Lytle moved that orders on the treasurer for six dollars ($6.00) each, which sums shall be deducted from the amount due the state society as "back assessments," shall be drawn in favor of those who have become or who may become eligible for reinstatement in this society in 1908, and such individuals are hereby declared reinstated in this society; but it is specifically provided that in each such case of reinstatement it must be shown by the books of the treasurer that the individual has paid "six dollars"

($6.00) into the society as State assessments for 1906 and 1907, and that in each case it must be shown by the lists received from the state treasurer that the individual so paying had been dropped from the rolls by the state officers in 1906, for non-payment of dues, and in each such case it must be shown that the individual has become eligible for reinstatement in 1908. Carried.

It was after 6 o'clock when the business session adjourned until 8.30 o'clock the same evening.

EVENING SESSION.—The vice-president Charles A. Wall, called the meeting to order at 8.30 p. m. During the temporary absence of the secretary, Dr. L. M. Francis was appointed secretary pro tem.

The following amendments to the by-laws were then presented by Dr. A. T. Lytle, and, under the rules, were ordered to lie on the table until the next regular meeting: "Moved, that unanimous consent be granted under Section 2, Chapter 15, to the suspension of Section I, Chapter 15 of the by-laws."

Be it *Resolved,* That Section I, Chapter 12 of the by-laws be so amended as to read: "Each member shall pay annually the sum of two dollars ($2.00) which shall be due on the first day of January of the current year. At the same time he shall pay the amount of the per capita State assessment fixed by the House of Delegates for the current year."

*Resolved,* That Chapter II be amended by adding a new section as follows: "Sec. 12. Honorary members may be elected at any regular meeting provided their names have been represented at a previous regular meeting and recommended by the council, and such action is the unanimous vote of twenty (20) members present. Their dues shall be paid by the Society."

*Resolved,* That Sec., 7, Chapter VI be amended to read as follows: "Clerical and legal services, when necessary, shall be furnished by the council."

Be it *Resolved,* That Par. 1, Sec. 4, Chap. IV of the by-laws shall be so amended as to read: "The treasurer shall be the custodian of all the funds of the society and he shall collect all county dues, State assessments and any other moneys due the society. He shall at once place all such funds received by him in a depository to be designated by the council. He shall pay out no moneys except on order of the council, counter signed by the president and the secretary, unless otherwise provided for in these by-laws."

Be it *Resolved* That Section 2, Chapter 12 of the by-laws shall be so amended as to read:

"All members who have not paid their annual County dues and State assessments on or before the first day of April of each year, shall be placed in the list of members in arrears for dues and assessments, and so reported in the society at its next meeting. Members shall not be entitled to receive the publications, or defense for alleged malpractice until all their dues and assessments are paid."

Be it *Resolved* That Par. 2, Section 4 of Chapter 4 of the by-laws shall be so amended as to read:

"He shall, on or before January 1st of each year, mail to each member a bill for his dues and assessments for the ensuing year. He shall, before the first of each of the months of April, May, June and December, notify all members of their arrears. He shall keep proper books of account, which shall be, at all times, open for examination by the Council. He shall make reports to the council when requested, and one to the society at its annual meeting."

Dr. Rochester asked if any special action had been taken against the anti-vivisection bill. He was informed that such had been done by the council.

Dr. Rochester then moved that the secretary be instructed to send to our representatives in the assembly a protest against the passage of the anti-vivisection bill. Carried.

Dr. E. E. Snow, of Batavia, president of the Eighth District Branch, then paid his final visit to the county society and addressed the meeting.

Among other things, he spoke of the coming annual meeting in Batavia, and expressed a wish to see as many as possible from this society in attendance.

This ended the business session and the literary part of the program was then carried out.

Five minute talks were given as follows: Thomas B. Carpenter, Albumen; Eugene Smith, Intussusception in children; George F. Cott, Probability of intracranial complications following middle ear infection; H. C. Rooth, Radical treatment of incipient hip-joint disease. Report of case; A. E. Woehnert, Casts; J. E. King, Influence of relaxed utero-sacrals on causation of symptoms in retroversion.

Each paper was very neat and gave, in a condensed form, the very best on the subject, as was shown by the marked interest in the presentations, and further by the active discussion which followed each talk.

Dr. Wm. C. Krauss then read a very interesting paper on "Diagnosis of Spinal Cord Tumors. Report of three cases seen within a period of ten days."

This paper was also listened to with marked interest, and discussed at its completion.

At the conclusion, a vote of thanks was tendered to all who presented papers and also to those who gave clinics in the morning.

The appreciation of the manner in which the program for this session had been gotten up by the special committee on program consisting of Drs. McKee, Grover Wende and Edward Clark, was shown by the marked attendance.

The committee divided the program into three parts:

Two clinics at the Buffalo General Hospital (one by Dr. Charles Cary at 10 a. m., and the other by Dr. Roswell Park at 11 a. m.)

The afternoon session, which was devoted entirely to business, and the evening session, devoted to scientific discussion.

The attendance was the best of any for a long time.

A hearty vote of thanks was accorded the program committee, whereupon adjournment followed.

